# Self-sufficiency in the healthcare workforce: a system dynamics model of the domestic and foreign educated nursing and midwifery workforce in Ireland

**DOI:** 10.1186/s12960-025-01004-4

**Published:** 2025-08-21

**Authors:** Terence Hynes, Paul Caulfield, Peter O’Connor, John Cullinan

**Affiliations:** 1 Department of Health, Block 1 Miesian Plaza, 50–58 Lower Baggot Street, Dublin 2, D02 XW14 Ireland; 2 Discipline of Economics, University of Galway, University Road, Galway, H91 TK33 Ireland; 3 Parliamentary Budget Office, Houses of the Oireachtas, Leinster House, Kildare St, Dublin 2, D02 XR20 Ireland

**Keywords:** System dynamics, Supply model, Nurses, Midwives, WHO code of conduct

## Abstract

**Background:**

World Health Organization (WHO) projections point to an increasing global demand for nurses and midwives, leading to shortages in many countries, particularly in less developed regions. Ireland, the context for this study, currently relies heavily on foreign educated nurses and midwives to meet its demand, with Government policy moving towards a domestic recruitment model. This paper estimates the recruitment requirement and associated nursing and midwifery student intake over time under different reform scenarios. It also highlights policy considerations for countries, like Ireland, aiming to comply with the WHO Code of Conduct on the International Recruitment of Health Personnel.

**Methods:**

This paper develops and applies a system dynamics model of the domestic and foreign educated workforce supply by age and gender and is based on regulatory data on stocks and flows from the national professional regulator for nurses and midwives. The model scope and design was informed by a problem statement developed in a series of workshops with officials in the Office of the Chief Nursing Officer. A range of scenario and sensitivity analyses are also undertaken.

**Results:**

In 2021, the base year of our projection horizon, we estimate that Ireland needed to recruit 3019 professionally active whole-time-equivalent (WTE) nurses and midwives. This would have required 3965 student places four years earlier in 2017 to meet this demand domestically. This is 153% higher than the 1570 student places that were available in that year. The recruitment requirement rises to 4497 by 2051, a 49% increase on 2021 levels. Foreign educated nurses and midwives, in terms of WTEs, start at 45% of projected demand in 2021 and range from 57% in the baseline scenario to 16% in the most ambitious reform scenario in 2051.

**Conclusions:**

The analysis suggests that Ireland requires a significant increase in nursing and midwifery student places to achieve self-sufficiency and that this will take time to achieve. Moreover, in addition to a sufficient domestic supply of nurses and midwives, self-sufficiency will also depend on managing demand volatility. Finally, countries anticipating a shift to a predominantly older population should ensure they have enough student places available before the demographic transition occurs to meet the associated health workforce requirements through the domestic education system.

## Background

World Health Organization (WHO) projections highlight an increasing global demand for nurses, with a global requirement for an estimated 36 million by 2030 and a shortage of 5.7 million, primarily in the African, South-East Asia, and Eastern Mediterranean regions [1]. Notwithstanding this maldistribution, a number of countries in the American, European, and Western Pacific regions are also projected to experience national shortages. The situation is similar for midwives, and the WHO estimates there will be a global shortage of 750,000 by 2030 [2].^1^ Shortages of nurses and midwives due to the migration of health workers from lower- to higher-income countries exacerbates the situation in the developing world. As a result, the WHO has developed a voluntary Code of Conduct on the International Recruitment of Health Personnel, which guides and supports countries to manage the international flows of health care professionals [1, 3]. The Code of Conduct was approved in the Sixty-third World Health Assembly Resolution WHA63.16, and is intended, inter alia, to promote principles and practices for the ethical recruitment of health personnel, to improve the legal and institutional framework required for recruitment, and to promote international discussion and cooperation on recruitment, with a particular focus on strengthening the health systems of developing countries.

The Irish health system is heavily reliant on the recruitment of foreign educated health workers, who play an essential role in enabling the continued delivery of healthcare in Ireland. In 2022, 49% of nurses in Ireland were foreign educated, the highest in the Organisation for Economic Co-operation and Development (OECD) [4]. In 2024, 78% of first-time registered nurses and midwives were educated abroad [5]. This reliance on foreign educated nurses and midwives is a function of the relatively low number of nurses graduating from Irish higher education institutions (HEIs). In 2021, the number of new nursing graduates was 23rd highest amongst OECD countries, at 32 per 100,000 [6]. Ireland had 2 midwifery graduates per 100,000 in 2022, 11th highest in the OECD [7].

The reliance on foreign educated health workers is occurring despite the demand for nursing and midwifery college courses significantly exceeding supply. For example, in 2021, there were 2032 first year places on offer in bachelor’s degree courses, compared to 5951 first-preference applications for such courses. However, interest amongst 15-year-old students in pursuing careers as nurses has been identified as a concern across a number of OECD countries, including Ireland [8]. Furthermore, looking forward, while total student enrolments in Irish HEIs are projected to increase from 196,005 to 239,655 over the period 2021 to 2031, they are projected to decline to 217,105 by 2041 [9]. This is partly driven by a declining number of potential students in the typical age cohorts and this will have implications for the future demand for nursing and midwifery college courses. Ireland had previously experienced an over-supply of nurses and midwives in the 1980 s due in part to the lack of available employment. Significant recruitment of foreign educated nurses and midwives began in the latter half of the 1990s [10].

Demand for health workers in Ireland is expected to grow into the future, in part because Ireland is ageing at a much faster rate than most other EU countries [11]. In addition, demand for nurses and midwives has demonstrated considerable volatility in recent decades. The number of first-time registrations^2^ in 2006 was 5174, which was 8% of the practising nursing and midwifery workforce in that year [13]. This declined to 2211 in 2010, or 3% of practising nurses and midwives, before rising to 4994, or 6% of professionally active nurses in 2021 [14–16]. This volatility in registrations reflects economic cycles, including the 2008/09 Great Recession, and in the latter period the COVID-19 pandemic [17]. For example, during the economic downturn, the Government of Ireland implemented a moratorium on the recruitment of staff to the public service, including the health service, with the intention of reducing public expenditure [18].

The key initiative driving strategic change in the Irish health system, including health workforce planning, is the Sláintecare reform programme, which has been set out in a series of reports [19, 20]. Nursing and midwifery care was also addressed by the Expert Review Body on Nursing and Midwifery, established by the Minister for Health in 2021 [21]. One recommendation outlined the need for the Department of Health to develop an integrated workforce strategy for nursing and midwifery, including the planning and forecasting of staffing requirements. In 2001, the Department of Health and Children undertook the first national level workforce planning exercise for nursing and midwifery and published guidance on the ethical recruitment of foreign educated nurses and midwives [10, 22].

Within this context, this paper aims to identify the optimal nursing and midwifery annual recruitment requirement for Ireland to become self-sufficient. Self-sufficiency, in this context, means having a sufficient supply of domestically educated nurses and midwives graduating each year to meet health system requirements. To achieve this aim, we develop and apply a workforce supply model based on a system dynamics modelling methodology that separately models the domestic and foreign educated workforce supply. This allows us to simulate how the health and care system can transition away from an over-reliance on the foreign educated workforce supply in the medium to long-term. The paper contributes to our understanding of how system dynamics modelling can support workforce planning and informs policymakers on factors that may need to be considered when examining policies to comply with the WHO Code of Conduct on the International Recruitment of Health Personnel.

## Methods

### Forecasting strategies

Workforce planning exercises can employ a number of different forecasting strategies, including supply-based, demand-based, need-based, and benchmarking approaches [23–25]. Since previous research in the Irish context has estimated nursing and midwifery demand for publicly funded acute hospitals [26], this paper uses a supply-based approach. The system dynamics methodology approach we adopt has previously been used for both demand and supply workforce projections across a range of country settings and Appendix 1 highlights a number of publications that have applied this methodology in this context, including three papers that have considered nurses [27–29].

### System dynamics modelling

System dynamics is a methodology and mathematical modelling technique used to frame, understand, and discuss complex issues and systems over time. System dynamics models are also widely applied as problem-solving tools, as they can be used to represent complex systems visually and to demonstrate how different qualitative factors relate and interact with each other, before numerical values are added. The ability to represent system dynamics models visually is a key feature that contributes to their applicability to public policy and the development of models that require input from a diverse range of stakeholders.

Stock and flow diagrams are the building blocks of system dynamics models.^3^ Mathematically, system dynamics models are defined by differential equations which represent inflows and outflows over time and are numerically solved through integration to identify the value of a stock at any given point in time [30]. This paper uses the software package Vensim Professional version 9.4.0 for model development and simulation.

### Data sources

The principal data source for this paper is the Nursing and Midwifery Board of Ireland (NMBI)^4^ register, which provides information on the number of registered nurses and midwives, the number of professionally active nurses and midwives, flows of individuals coming onto the register, and flows of individuals coming off the register. A professionally active nurse or midwife is one who is practising but not necessarily patient facing (i.e. may not be working in a clinical setting). This may include a small proportion of professionally active nurses and midwives working abroad. This database was chosen for the analysis due to its comprehensive coverage of the nursing and midwifery workforce, in contrast to that available from other sources, such as the national health service, which would only partially cover the profession. A recent reform of the database has substantially increased the quality and availability of this dataset, which was essential in enabling the development of this model.^5^ The NMBI require that all nurses and midwives pay an annual retention fee to maintain their registration and those who fail to pay are removed from the register. Data from all sources were collated and processed in Excel before being inputted into Vensim. Appendix B provides full details of the variables and parameters used in this paper.

### Model design

The model design process started with the development a ‘problem statement’ that represents the essential aspects of the problem or decision that needs to be made at the outset of the research process. The structure, flows, and stocks of the model outlined are then developed from this problem statement. In this paper, a problem statement for our model was identified through a workshop format with three officials in the Office of the Chief Nursing Officer of the Department of Health in Ireland. There were five workshops which served to identify factors influencing the nursing and midwifery workforce, to develop a problem statement, to communicate model concepts and design to officials, and to inform the sensitivity analysis. The problem statement developed was:

- Ireland is not educating enough nurses and midwives to meet domestic demand, which is leading to high levels of recruitment from overseas.
- There is a government commitment to move towards a domestic recruitment model, which entails educating the required number of nurses and midwives in Ireland and reducing the reliance on overseas recruitment.
- Demand for nurses and midwives is outstripping supply globally.
- Student places in Ireland are oversubscribed.

The model developed to address this problem statement divides the nursing and midwifery workforce into four distinct stocks according to gender and whether they were educated domestically or abroad. These are modelled in terms of whole-time-equivalent (WTE) staff and relate to the number of professionally active nurses and midwives, rather than the greater number that are licenced to practise. Parameters for the three outflows of these stocks—retirements, emigration, and net attrition—were defined based on exits of those licenced to practise from the register by age. Appendix B documents the key parameter values for the stocks and flows of the model.

**Fig. 1:**
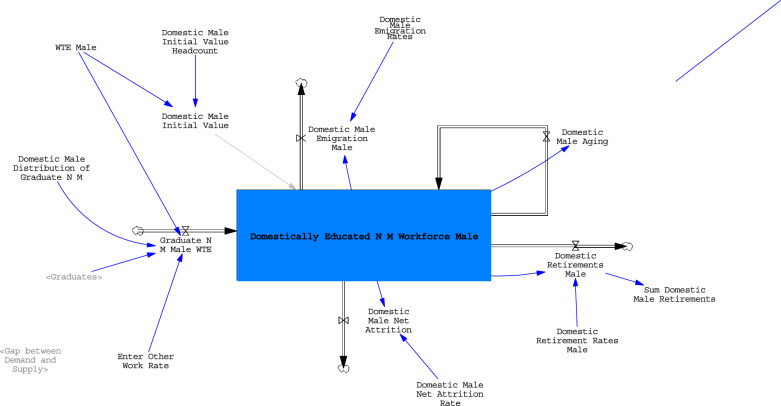
Image of model stocks and flows for male, domestically educated nurses and midwives

Demand is modelled in the aggregate with the growth rate based on published estimates which include, inter alia, the effects of population growth, changes in the age structure, healthy ageing, and changes in service delivery [26]. The gap between demand and supply in any period is used to determine the number of foreign educated nurses and midwives added to the model. If the gap is zero, none are added. If it is non-zero, the model adds a commensurate number of foreign educated nurses and midwives. This reflects recruitment practices at organisational level in the real world and was based on expert input from the workshops. Figure 1 presents an example image of the model, showing stocks and flows for ‘male, domestically educated nurses and midwives’ and its various inflows and outflows. Diagrams of the higher education system and the demand projections are included in Appendix C.

The key output from the model is the recruitment requirement, which is the number of new professionally active nurses and midwives that need to be added to the workforce each year to ensure demand meets supply. It is defined as the total of so-called ‘expansion demand’ and ‘replacement demand’. Expansion demand is the number of new nurses and midwives required each year to meet demand, while replacement demand is the aggregate of all outflows from the model—net attrition, emigration, and retirement in each year [31, 32]. It reflects the number of nurses and midwives that need to be replaced each year.

### Scenario analysis

The year 2021 was chosen as the starting year for the model, as it was the most recent year with available data from the NMBI. The projection period is 30 years to allow for the long-term effects of polices on the make-up of the workforce to be assessed. Three separate scenarios are considered and, for all, demand grows at a compound annual growth rate of 1.4% [26]. Scenario A reflects a baseline projection with no increase in student places. In contrast, Scenarios B and C increase the numbers of student places, such that nursing and midwifery graduates, measured in terms of WTEs, equal the recruitment requirement in a given year. Scenario B does this rapidly (by 2030), whereas Scenario C represents a slower adjustment (by 2040).

### Sensitivity analysis

A sensitivity analysis is undertaken for two variables where there is relatively more uncertainty surrounding their future values, i.e. the growth rate in projected demand and net attrition. Their effect is examined in terms of both projected demand and the recruitment requirement and a 15% ± change in the baseline parameter is simulated.

## Results

**Fig. 2:**
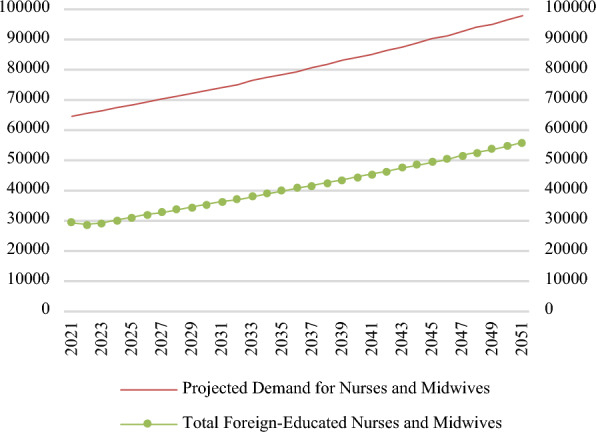
Stock of nurses and midwives in the baseline scenario (WTEs)

Figure 2 shows that overall demand (stock) starts at 64,383 nurses and midwives and grows by 33,321 to 97,704 by 2051, representing a 52% increase. In this scenario the student intake remains at its 2021 value, so that the increased demand is met through additions of foreign educated nurses and midwives to the workforce. The number of foreign educated nurses and midwives starts at 29,285 in 2021 and grows by 26,354 to 55,639 in 2051, representing an 89% increase. At baseline, foreign educated nurses and midwives in WTE are 45% of projected demand, and by 2051, represent 57% of projected demand.

**Fig. 3:**
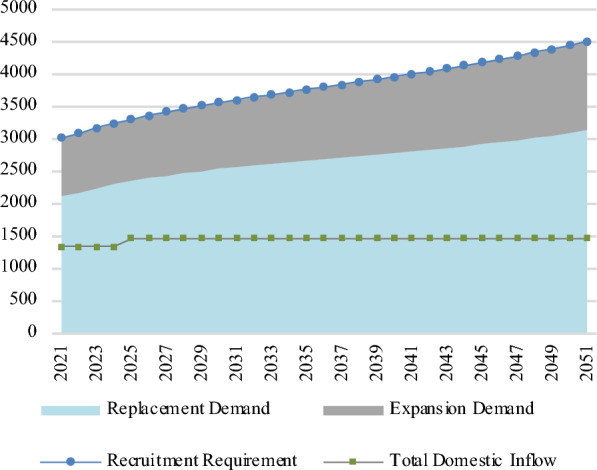
Flows of nurses and midwives in the baseline scenario (WTEs)

In terms of flows, Fig. 3 shows that as of 2021, the recruitment requirement was 3019 nurses and midwives. This rises to 4497 by 2051, a 49% increase. Under the baseline scenario, replacement demand is approximately 70% of the recruitment requirement throughout the projection horizon. The number of domestic graduates in terms of WTE that go on to practise as a nurse or midwife each year is defined as total domestic inflow. This is approximately 1,469 throughout the projection horizon.

**Fig. 4:**
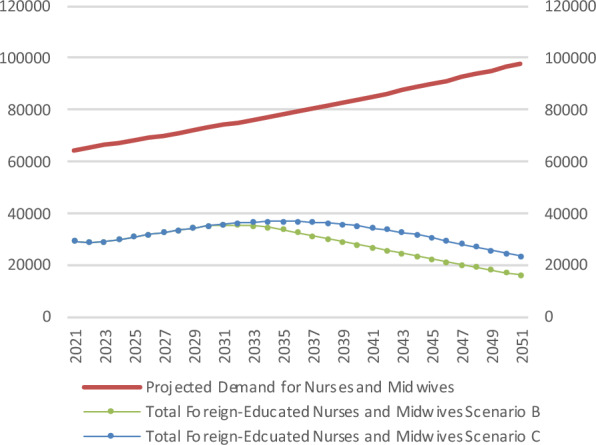
Stock of nurses and midwives in the policy scenarios B and C (WTEs)

Figure 4 presents the stock view for Scenarios B and C, which both increase domestic graduate output over varying time periods. For Scenario B, after a brief rise to a peak of 35,293 in 2032, the number of foreign educated nurses and midwives falls to 16,080 by 2051. This represents 16% of projected total demand in that year. In Scenario C, foreign educated nurses and midwives rises to 36,700 in 2035, and then falls to 23,374 in 2051, which is 24% of domestic demand.

**Fig. 5:**
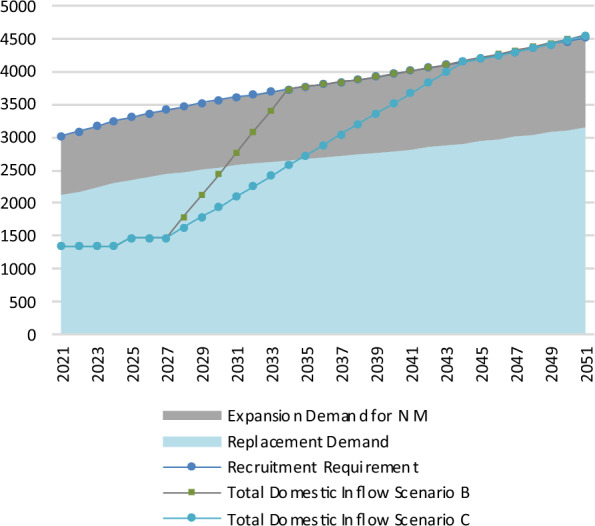
Flows of nurses and midwives in policy scenarios B and C (WTEs)

Figure 5 shows the flow view of Scenarios B and C. Student places rise from 2024 onwards, though it takes four years for this to be observed in total domestic inflow. The point at which foreign educated nurses and midwives are no longer being recruited in Ireland occurs when the total domestic inflow equals the recruitment requirement. In Scenario B, the total domestic inflow rises to equal the recruitment requirement by 2034, at a level of 3729. For Scenario C, total domestic inflow equals the recruitment requirement by 2044, at a level of 4143.

Table 1 presents the sensitivity analyses relating to the growth rate in projected demand and net attrition. This analysis focuses on projected demand and the recruitment requirement, where each parameter is increased/decreased by 15% above/below its value in the baseline scenario. In relation to projected demand, a 15% increase in the baseline growth rate increases demand by 6% to 103,960, whereas a 15% decrease reduces it by approximately the same percentage to 91,812. The percentage change for the recruitment requirement is ± 10%. The recruitment requirement is impacted by the growth rate through an increase in both expansion demand and replacement demand. For replacement demand, an increase in the number of nurses and midwives results in an increase in the value of this variable because it is calculated based on fixed percentages for attrition, emigration, and retirement. Finally, for net attrition, an increase in this variable increases outflow for the supply of nurses and midwives. This leaves demand unaffected but impacts the recruitment requirement through its impact on replacement demand. The percentage change for net attrition is approximately 3%.

**Table 1:** Sensitivity analysis

		Baseline scenario	
		Demand in 2051	Recruitment requirement in 2051
		97,704	4497
Input parameter adjusted	% Change in baseline parameter	Change in demand (%)	Change in recruitment requirement (%)
Demand growth rate	+ 15%	103,960 (+ 6.4%)	4947 (+ 10%)
Demand growth rate	− 15%	91,812 (− 6%)	4085 (− 10%)
Net attrition rate	+ 15%	97,704 (0%)	4627 (+ 3%)
Net attrition rate	− 15%	97,704 (0%)	4367 (− 3%)

## Discussion

The results highlight that Ireland is significantly undersupplying domestic nursing and midwifery graduates. The recruitment requirement in WTE in 2021 was 3019, which would require 3495 graduates or 3965 student places (4 years earlier) to meet this demand. This represents 153% of the 1570 student places that were available in 2017 and suggests significant increases in nursing and midwifery student intake are urgently needed if Ireland is to reach self-sufficiency in a timely manner. The reform scenarios identify that by between 2034 and 2044, Ireland will need between 3729 and 4143 graduates in terms of WTEs a year. These results reflect both the growing demand for healthcare due to, for example, demographic growth and aging, as well as the historic undersupply of domestically educated nurses and midwives. The sensitivity analysis suggests that the outer bound may be approximately 3% above or below the recruitment requirement by the end of the projection period. This would require between 4895 and 5439 students over the period 2030 and 2044.

The estimated demand for students in this paper also raises questions about the feasibility of attracting that many students to fill the places available. Data from the Central Applications Office (CAO) in Ireland show that 4,500 students ranked a nursing or midwifery course as their first preference in their applications for higher education in 2021. The CAO processes applications for undergraduate courses in Irish HEIs. Decisions on admissions to undergraduate courses are made by the HEIs who instruct the CAO to make offers to successful candidates. This suggests that there is significant capacity to expand student places between the period 2030 and 2036. The CAO application system operates on a demand and supply basis, meaning that as more student places are available the entry requirements in terms of points in the final year examinations will fall. However, the extent to which this is desirable requires consideration. In addition, a challenge arises due to underlying demographics, which will mean fewer people in the 18–26 age category in the future. However, as noted previously, declining interest amongst students to become nurses is a complicating factor [9]. This implies that countries should ensure they have sufficient student places before their demographic transition to a society with relatively more older people. The Expert Review on Nursing and Midwifery has highlighted the potential of alternative routes into the nursing and midwifery career such as, for example, an apprenticeship model that offers the possibility of increasing the pool of potential nurses and midwives outside the population cohorts that typically attend college [21].

Demand volatility has been historically high in Ireland due to economic cycles and the COVID-19 pandemic. A reliance on foreign recruitment allowed rapid increases and decreases in the number of foreign educated nurses and midwives joining the labour force. In a future where Ireland’s annual recruitment requirement is composed wholly of those from domestic sources, this flexibility will not be possible. This is because, firstly, it takes four years for someone to graduate from a degree, secondly, the capability to anticipate economic cycles and pandemics is limited to less than 12 months ahead, and thirdly, the capacity of the higher education system in a country is relatively inflexible, meaning significant, rapid increases or decreases in student intake are challenging.

Overall this implies that there may be periods where the actual effective demand for nurses and midwives in any given year is higher or lower than graduate output. As such, achieving self-sufficiency in supply requires other policy measures beyond having an adequate number of graduates. Medium-term budget planning would expand the horizon over which public health system planning could be undertaken. Focusing on more regular moderate increases in health system resources within budgets would minimise the requirement for rapid increases and decreases in workforce intake. Other research has highlighted the importance of policies which enable surge capacity in the workforce in response to demand shocks, such as pandemics [33]. It may also be appropriate not to aim for total self-sufficiency in domestically educated graduates relative to the annual recruitment requirement. A certain amount of migration into a country occurs for reasons unrelated to undersupply of domestic graduates and, as such, there may be a natural inward migration from other countries. Furthermore, there are benefits which may accrue to health systems in developing regions from migration of staff, where they either return home with expanded skillsets or are in a position to return remittances. Lastly, there is inherent uncertainty in projection exercises which means accurately estimating demand is challenging. The WHO Code of Conduct provides guidance in formulating and implementing bilateral agreements and other legal instruments to support member states in ethically managing the recruitment of health personnel [3].

### Strengths and limitations

In the application of system dynamics modelling to this exercise, the diagrammatic feature of the modelling software easily communicated the model structure to officials in the Office of the Chief Nursing Officer in the context of a workshop. Model development was rapid, and the data requirements are likely available across a number of other jurisdictions that regulate the nursing and midwifery professions. Grounding the model design in a problem statement also allowed for a clear articulation of how the model would be used to support workforce planning decisions. System dynamics modelling is promoted as a method that can be utilised in a ‘real world policy context’; this was true in this case, though the timelines involved in this project meant that the focus quickly turned from the problem statement and defining model-boundaries, to producing model results. For this reason, causal loops were not formally embedded in the model’s design.

Historic time series data which would have allowed for model calibration were not available pre-2021. Moreover, nurses and midwives are grouped together for the purpose of our analysis. This is necessary to maintain model simplicity and because of limitations in our ability to distinguish between those individuals that are operating in nursing or midwifery roles. Pre-2011, a degree qualified individuals to practise as both a nurse and a midwife. Another limitation is that there are likely to be distinct demand growth rates for nurses and midwives. Certain model parameters, in particular net attrition, are subject to a high degree of uncertainty and are more challenging to parameterise with available data. The results of the paper are sensitive to the future evolution of retention and attrition in the workforce.

## Conclusion

Ireland requires a significant increase in nursing and midwifery student places. As a result, there will be significant timelines involved in Ireland reaching self-sufficiency. Moreover, self-sufficiency in supply depends in part on managing demand volatility, as well as having a sufficient domestic supply of nurses and midwives. Finally, countries anticipating a shift to a predominantly older population should ensure they have enough student places available before the demographic transition occurs to meet the associated health workforce requirements through the domestic education system.

## Figures and Tables

**Fig. 6:**
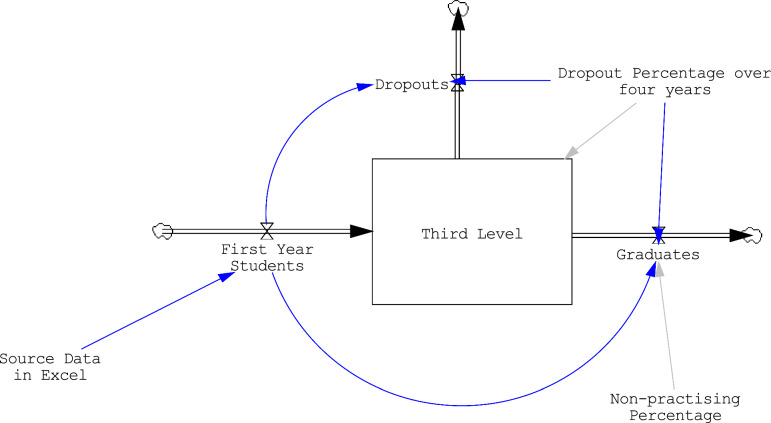
Image of higher education system

**Fig. 7:**
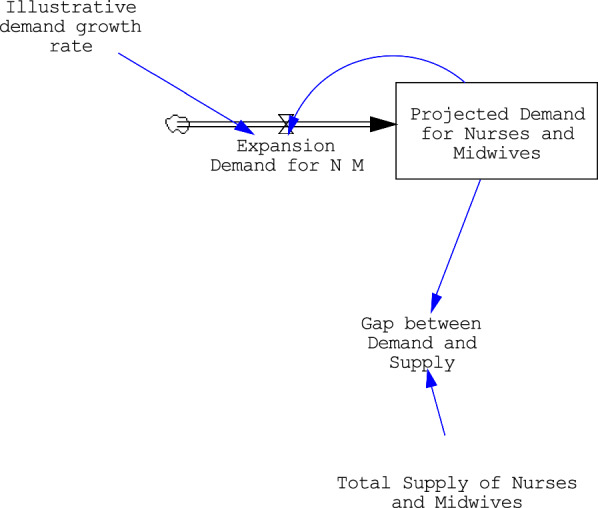
Image of demand projections

## Data Availability

Data and sources are provided with in Table 3 of Appendix B in the manuscript except where three cells are redacted to prevent statistical disclosure due to small cell size.

## Appendix A

See Table 2

**Table 2:** Examples of previous workforce planning models employing a system dynamics methodology

Year	Title	Country setting	Workforce focus	References
2010	Forecasting the need for medical specialists in Spain: Application of a system dynamics model	Spain	Doctors	[34]
2010	A dynamic forecasting model for nursing manpower requirements in the medical service industry	Korea	Nurses	[28]
2013	Forecasting the absolute and relative shortage of physicians in Japan using a system dynamics model approach	Japan	Doctors	[35]
2013	The number of Japanese radiologic technologists will be increased in 40 years	Japan	Radiologic Technologist	[36]
2014	Modelling the future of the Canadian cardiac surgery workforce using system dynamics	Canada	Doctors	[37]
2015	Future requirements for and supply of ophthalmologists for an aging population in Singapore	Singapore	Doctors	[38]
2015	Forecasting future needs and optimal allocation of medical residency positions: the Emilia-Romagna Region case study	Italy	Doctors	[39]
2015	How many dentists does Sri Lanka need? Modelling to inform policy decisions	Sri Lanka	Dentists	[40]
2018	A supply model for nurse workforce projection in Malaysia	Malaysia	Nurses	[29]
2019	Projecting future supply and demand for physical therapists in Japan using system dynamics	Japan	Physical Therapists	[41]
2020	Projecting supply and demand for pharmacists in pharmacies based on the number of prescriptions and system dynamics modelling	Japan	Pharmacists	[42]
2021	Nursing manpower forecast for cancer patients	Taiwan	Nurses	[27]

## Appendix B

See Table 3

**Table 3:** Baseline parameter values and sources

Name	Gender	Location of education	Data source	Baseline values for variables									
Student dropout percentage over four years	Both	NA	Higher Education Authority [43]	0.111									
Percentage of graduates that become professionally active			NMBI	0.91409									
WTE male			HSE [44]	0.94									
WTE female			HSE [44]	0.85									
Demand growth rate			ESRI	1.4									
				**20–24**	**25–29**	**30–34**	**35–39**	**40–44**	**45–49**	**50–54**	**55–59**	**60–64**	**65 + **
Age on first registration	Male	Foreign	NMBI	0.02	0.07	0.06	0.02	0.01	0.00	0.00	0.00	0.00	0.00
Age on first registration	Female	Foreign	NMBI	0.07	0.32	0.26	0.10	0.04	0.02	0.01	0.00	0.00	0.00
Age of graduates on first registration	Female	Domestic	NMBI	0.65	0.10	0.07	0.04	0.03	0.02	0.01	0.00	0.00	0.00
Age of graduates on first registration	Male	Domestic	NMBI	0.06	0.01	0.01	0.00	0.00	0.00	0.00	0.00	0.00	0.00
Emigration rates	Male	Domestic	NMBI	*	0.03	0.00	0.00	0.00	0.00	0.00	0.00	0.00	0.00
Emigration rates	Female	Domestic	NMBI	*	0.03	0.00	0.00	0.00	0.00	0.00	0.00	0.00	0.00
Emigration rates	Male	Foreign	NMBI	0.03	0.03	0.00	0.00	0.00	0.00	0.00	0.00	0.00	0.00
Emigration rates	Both	Foreign	NMBI	0.03	0.03	0.00	0.00	0.00	0.00	0.00	0.00	0.00	0.00
Net attrition rate	Both	Foreign	NMBI	0.00	0.00	0.01	0.02	0.02	0.01	0.02	0.03	0.00	0.00
Net attrition rate	Both	Domestic	NMBI	0.00	0.00	0.02	0.02	0.02	0.02	0.02	0.04	0.00	0.00
Retirement rates	Both	Domestic	NMBI	0.00	0.00	0.00	0.00	0.00	0.00	0.00	0.00	0.11	0.31
Retirement rates	Male	Foreign	NMBI	0.00	0.00	0.00	0.00	0.00	0.00	0.00	0.00	0.11	0.28
Retirement rates	Female	Foreign	NMBI	0.00	0.00	0.00	0.00	0.00	0.00	0.00	0.00	0.11	0.28
No. of nurses and midwives headcount	Male	Domestic	NMBI	124	268	287	396	385	383	275	307	223	97
No. of nurses and midwives headcount	Female	Domestic	NMBI	1885	4257	4805	4919	4341	5002	4183	4679	3007	1182
No. of nurses and midwives headcount	Male	Foreign	NMBI	*	286	1219	1008	494	681	616	257	132	31
No. of nurses and midwives headcount	Female	Foreign	NMBI	121	2856	5419	3948	4248	4733	3866	2066	1360	603

*Cell suppressed to prevent statistical disclosure

## Appendix C

See Figs. 6, 7

## Footnotes

1 In Ireland, midwifery is a separate and distinct profession from nursing, according to the Nurses and Midwives Act 2011.

2 First-time registrations are those nurses and midwives that have just graduated from Irish HEIs or those joining from abroad, and is a useful measure of the annual demand for new nurses and midwives. As such, in periods of economic downturn, it is likely that the registrants from domestic HEIs may not go on to join the nursing and midwifery workforce in Ireland. For example, in 2010, a year of economic contraction, 75% of nursing graduates were no longer recorded in state administrative datasets in Ireland [12]. A smaller, but unknown proportion, were likely not employed as nurses or midwives at all.

3 Stock variables describe the states or amounts of the system, while flow variables depict the rates of change—increases or decreases—of stocks. Therefore, stocks accumulate or deplete in a model.

4 The NMBI is the independent, statutory organisation which regulates the nursing and midwifery professions in Ireland. Since it is not possible to accurately distinguish those working in nursing or midwifery roles in the register at present, they are modelled collectively in this paper.

5 The NMBI's'Project Nightingale'delivered a modernised integrated online registration system, which provides an accurate and secure register, accessible data at every step of the registration process, improved records, streamlined processes, and enhanced self-service for applicants and registrants. See https://www.nmbi.ie/Registration.
